# Treatment outcome of class II malocclusion therapy including extraction of maxillary first molars: a cephalometric comparison between normodivergent and hyperdivergent facial types

**DOI:** 10.7717/peerj.14537

**Published:** 2022-12-12

**Authors:** Johan Willem Booij, Marta Fontana, Marco Serafin, Rosamaria Fastuca, Anne Marie Kuijpers-Jagtman, Alberto Caprioglio

**Affiliations:** 1 Gorinchem, The Netherlands; 2 Varese, Italy; 3 Department of Biomedical Sciences for Health, University of Milan, Milan, Italy; 4 Faculty of Dentistry, Universitas Indonesia, Jakarta, Indonesia; 5 Department of Orthodontics and Dentofacial Orthopedics, University of Berne, Berne, Switzerland; 6 Department of Orthodontics, University of Groningen, Groningen, Netherlands; 7 Department of Biomedical, Surgical and Dental Sciences, University of Milan, Milan, Italy; 8 Fondazione IRCCS Cà Granda, Milan, Italy

**Keywords:** Class II malocclusion, Normodivergency, Hyperdivergency, Maxillary first molar extraction, Cephalometric analysis

## Abstract

**Background:**

The dentoalveolar component of a Class II division 1 malocclusion can be orthodontically treated either with extractions or by distalization of the molars. This study aimed to compare skeletal, dentoalveolar and profile changes in normodivergent and hyperdivergent Class II Division I growing patients orthodontically treated with fixed appliances including maxillary first molar extraction.

**Methods:**

Sixty-four patients treated orthodontically with full fixed appliances including maxillary first molar extractions were retrospectively analyzed. Patients were divided into a normodivergent group (Group N; 30° ≤ SN^GoGn < 36°) consisting of 38 patients (17M, 21F; mean age 13.2 ± 1.3 years) and a hyperdivergent (Group H; SN^GoGn ≥ 36°) including 26 patients (12M, 14F; mean age 13.7 ± 1.1 years). Lateral cephalograms were available before (T0) and after treatment (T1) and cephalometric changes were calculated for 10 linear and 13 angular variables. The Shapiro–Wilk test confirmed a normal distribution of data, hence parametric tests were employed. The Student t-test was used to compare groups at baseline. The paired t-test was used to analyze intragroup changes between timepoints, and the Student t-test for intergroup comparisons. The level of significance was set at 0.05.

**Results:**

The Class II division 1 malocclusion was successfully corrected, and the facial profile improved both in normodivergent and hyperdivergent patients. Divergency increased by 0.76 ± 1.99° in Group N (*p* = 0.02) while it decreased −0.23 ± 2.25° (*p* = 0.60); These changes were not significant between groups after treatment (*p* = 0.680). Most dentoskeletal measurements changed significantly within groups but none of them showed statistically significant differences between groups after treatment. Dental and soft tissue changes were in accordance with the biomechanics used for this Class II orthodontic therapy.

**Discussion:**

The effect of orthodontic treatment of Class II division 1 malocclusion including extraction of the maxillary first molars in growing patients can be considered clinically equivalent in normodivergent and hyperdivergent patients. For this reason, this orthodontic treatment can be considered a viable option in the armamentarium of the Class II Division I therapy for both facial types.

## Introduction

Angle Class II Division I malocclusion is the most frequently treated malocclusion, with a reported prevalence of about 17.5% in America, Asia, and Europe and a mean prevalence in Africa of 6% (De Ridder et al., 2022). Thereby, its correction is a common procedure in orthodontic practice. Correction of the malocclusion can affect soft tissue harmony, depending on the overjet and its interaction with the facial profile (Janson et al., 2016). Class II malocclusion forms a heterogeneous group, in which the etiology is frequently multifactorial and where dentoalveolar and skeletal components are involved (McNamara, 1981). For these reasons, a correct diagnosis to decide upon the most appropriate treatment plan should consider not only the interarch molar relationships but—amongst others—also skeletal discrepancy, age, and patient compliance (Männchen et al., 2022).

Although the skeletal component of a Class II malocclusion can be treated by functional devices during childhood (Batista et al., 2018; D’Antò et al., 2015) or orthognathic surgery during adulthood (Pancherz et al., 2004), the correction of the dentoalveolar component can be carried out by two different approaches: maxillary molar distalization or extraction treatment. One of the most used procedures of maxillary molar distalization is headgear treatment that produces a significant distalization in children with anterior crowding (Sambataro et al., 2017). A non-compliance intraoral distalization device like the Hilgers’ pendulum is also used for molar distalization and control of the occlusal plane (Serafin et al., 2021) on par with skeletal distalization devices (Mohamed, Basha & Al-Thomali, 2018). Considering the Class II cases that are appropriate for extraction treatment, it is necessary to make a proper and careful space analysis in order to decide which pattern of extraction is the best for each patient. If the lower incisors have an adequate inclination over symphyseal bone, the mandibular crowding is minimal and the curve of Spee is not excessive, maxillary first premolar extraction can be considered (Vaden, Williams & Goforth, 2018). This treatment, called orthodontic camouflage, establishes extraction of maxillary first premolars to reach the retraction of the upper anterior teeth and the correction of the overjet with fixed appliances. The molars will move to a complete Class II and the canines in Class I relationship (Nangia & Darendeliler, 2001).

Another approach to Class II treatment involves extraction of the maxillary first permanent molars. Williams (1979) suggested this procedure to achieve a Class I occlusal relationship between the maxillary second molars and mandibular first permanent molars. This treatment strategy may be a good alternative in patients presenting with heavily decayed or extracted maxillary first permanent molars. Other reasons to take in consideration the extraction of the maxillary first molars are control of the vertical dimension in high-angle cases by extraction of teeth in the posterior part of the dental arch which has a bite-closing effect (Subtelny & Sakuda, 1964), and the extensively debated belief that posterior extractions would have less influence on soft tissue than premolar ones (Stalpers et al., 2007). Furthermore, extraction of the first molars may have a favorable effect on the inclination of the erupting second and third molar (Bayram, Ozer & Arici, 2009; Livas et al., 2011).

Booij et al. (2022) illustrated the way overjet correction and extraction space closure are achieved following maxillary first molar extraction. The approach described allows Class II correction without special precaution to preserve anchorage, largely eliminating the need for cooperation (Booij, Kuijpers-Jagtman & Katsaros, 2009). Data regarding the extraction of first maxillary molars as part of a comprehensive orthodontic treatment is limited. Premolar extraction is frequently accepted by patients and orthodontists, but extraction of healthy maxillary first molars is a difficult decision that may cause confusion and resistance of the parents and the referring dentist. However, the absence of laboratory costs, minimal negative impact on facial esthetics and low demand for compliance make extraction of the maxillary first molars a feasible alternative for Class II Division I therapy (Booij et al., 2011) if the third molar buds are radiographically present and the size and shape of the second and third molars is normal (Booij, Kuijpers-Jagtman & Katsaros, 2009; Booij et al., 2021).

In hyperdivergent Class II patients, the mandible is retropositioned in relation to the maxilla, affecting facial esthetics due to a convex profile, chin hypoplasia and gingival smile. Therefore, a treatment that reduces the lower facial height and moves the chin forward by counterclockwise mandible rotation is desired (Wang et al., 2022). Different strategies to achieve this have been proposed. These include the extraction planning to move molars forward and decrease the “wedge-type effect” in contrast with hypodivergent patients where nonextraction treatment is believed to favor vertical increase (Gkantidis et al., 2011). Although there are several studies regarding the control of the vertical dimension by premolar extraction (Kouvelis et al., 2018), the vertical changes in Class II therapy including maxillary first molars extraction draw less attention.

The present investigation was carried out to cephalometrically compare skeletal, dentoalveolar and profile changes produced by orthodontic treatment with fixed appliances including first maxillary molar extraction in growing subjects with a normodivergent *vs* hyperdivergent facial type. The null hypothesis was that there are no differences in treatment outcome between the two initial vertical growth patterns.

## Materials and Methods

### Sample

The protocol of this retrospective study was reviewed and approved by the Ethical Committee of the University of Milan, Italy (approval number ROS18/02) and the University Medical Center Groningen (approval number METc 2020/460). All the procedures followed the World Medical Organization Declaration of Helsinki. All patients gave written permission and signed informed consent for their anonymized data to be used.

*A priori* sample size calculation by Cohen’s equation was performed following d = 0.7, β = 80, α = 0.05; a minimum sample size of 26 patients per group was required. A sample of 100 patients consecutively treated with first maxillary molar extractions was obtained from a single orthodontic office. All patients were treated by a one clinician (J.W.B.) and selection criteria were the following: Caucasian, skeletal Class II malocclusion, bilaterally a full cusp Class II molar occlusion or end-to end; both sexes at late pubertal growth spurt (stage CS3 or CS4 according to the cervical vertebral maturation method) (McNamara & Franchi, 2018); skeletal divergency (SN^GoGn) larger than or equal to 30°. Exclusion criteria included: extracted or missing teeth except for the first maxillary molars, absence of maxillary third molars, not compromising medical conditions or craniofacial anomalies, and poor quality cephalograms at T0 or T1.

Thirty-six subjects were excluded from the original sample due to the inclusion criteria, thus the final sample consisted of 64 patients. These patients were divided into two groups based on the skeletal divergency calculated as SN^GoGn angle: group N (normodivergent; 30° ≤ SN^GoGn < 36°) and group H (hyperdivergent; SN^GoGn ≥ 36°). Group N consisted of 38 patients (17M, 21F; mean age 13.2 ± 1.3 years) and Group H of 26 patients (12M, 14F; mean age 13.7 ± 1.1 years).

**Figure 1:**
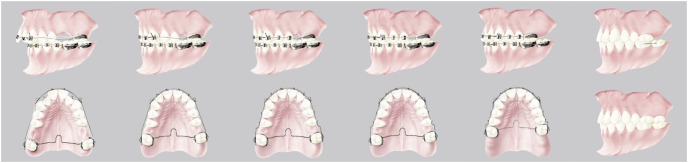
Graphic representation of the biomechanics used divided into three steps: Class II correction, space closure and torque, and detailing and finishing.

All patients underwent maxillary first molar extractions and were treated with fixed appliances with low-friction brackets, thin round wires and a palatal bar; the vertical control in hyperdivergent patients was provided by using a low palatal bar. The biomechanical method has been described previously (Booij, Kuijpers-Jagtman & Katsaros, 2009; Booij et al., 2022) and is shown in Fig. 1.

**Figure 2:**
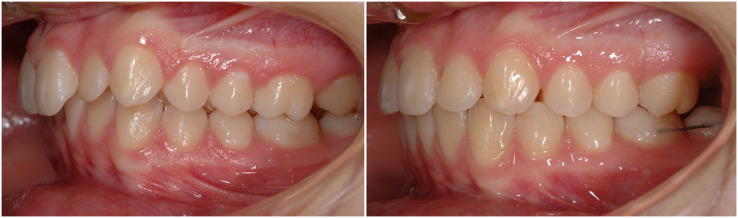
A case-report representing the initial and final occlusion after the treatment; note the post-treatment retention between lower first and second molar awaiting the upper third molar eruption.

The treatment is characterized by three subsequent phases: Class II correction, space closure and torque, and detailing/finishing. The characteristic occlusion at the end of the treatment is showed in Fig. 2. The mean total treatment time for both groups was 2.5 ± 0.6 years.

### Cephalometric measurements

**Figure 3:**
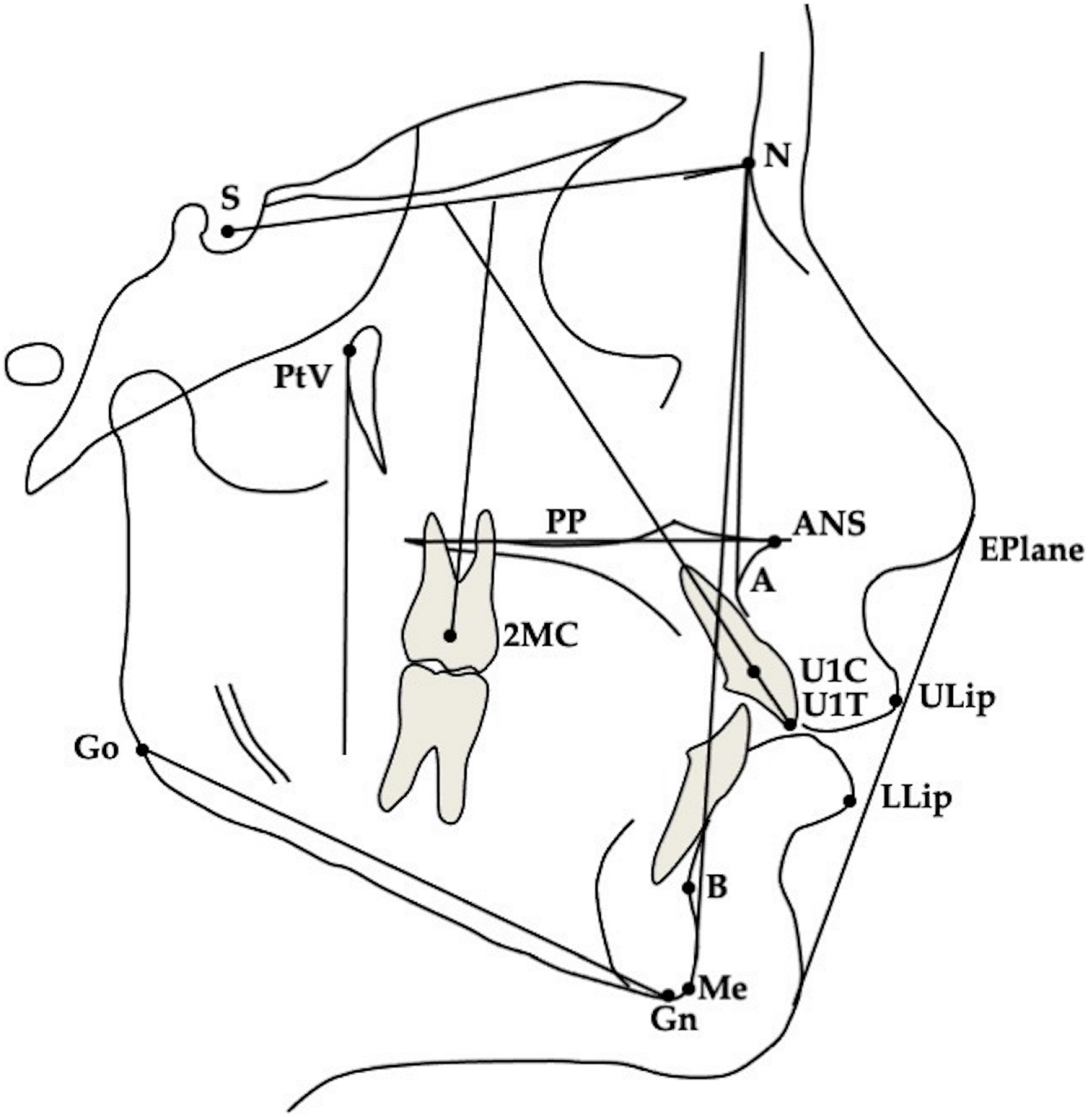
Cephalometric landmarks. S, Sella; N, Nasion; PtV, Pterygoid Vertical; Go, Gonion; Me, Menton; A, A point; B, B point; Eplane, Esthetic Plane; PP, Palatal Plane; ANS, Anterior Nasal Spine; ULip, Upper Lip; LLip, Lower Lip; U1C, Upper central incisor Centroid; U1T, Upper central incisor Tip; 2MC, second Molar Centroid.

For all patients lateral cephalograms were collected before (T0) and at the end of treatment (T1). The magnification factor was similar for all radiographs (approximately 8%), so no correction for magnification was performed in the analysis of the X-rays. One observer (M.S.) traced all radiographs and anatomical outlines, and landmark positions were verified by a second observer. When observers disagreed, the structure in question was retraced until consensus was reached. For bilateral structures, a single averaged tracing was made. A total of 23 measurements, 10 linear and 13 angular, was analyzed (Fig. 3).

### Method error and data analysis

The method of moments (MME) variance estimator was used to quantify the method error for both linear and angular measurements. Cephalometric tracings were repeated by the same observer for 12 subjects randomly selected from the two groups. The mean error and 95% confidence intervals (CIs) between the repeated measurements were calculated using the MME variance estimator. The mean intra-observer measurement error for the linear measurements was 0.47 mm (range 0.3–0.6 mm), and 1.0° for the angular measurements (range 0.5–1.3°).

The statistical analysis was carried out with SPSS software, version 22.0 (SPSS® Inc., Chicago, IL, USA). The Shapiro–Wilk test revealed normal distribution of the data, therefore parametric tests were employed. The Student t-test was used to compare the initial data of the two groups. For each group the means and standard deviations were calculated. The paired sample t-test was applied to analyze intragroup changes between T0 and T1 while the Student t-test was used for intergroup comparison.

## Results

*A posteriori* Cohen’s sample size calculation was performed to verify the effect size by the means and SD of SN^GoGn values; the result reported a d = 0.46 compatible with a medium effect size.

### Intergroup comparison at T0

Descriptive statistics for the cephalometric variables at T0 for Group N and Group H are presented in Table 1. Statistical analysis showed some significant between group differences in the initial data for the two groups. Regarding skeletal parameters, SNB was significantly larger for Group N with a measure of 75.58 ± 2.83° compared with Group H with a measure of 73.46 ± 2.15° (*p* = 0.002). SN^GoGn also differed significantly between groups (*p* = 0.001) with a larger angle in the Group H. Both soft tissue measurements were significantly different between groups at T0.

**Table 1:** Intergroup comparison between normodivergent (Group N) and hyperdivergent (Group H) groups at T0.

	Group N		Group H		*p*
Measurement	Mean	SD	Mean	SD	
**Skeletal**					
SNA (°)	83.04	3.16	81.62	2.53	0.061
SNB (°)	75.58	2.83	73.46	2.15	0.002*
ANB (°)	7.47	1.84	8.17	2.04	0.163
SN^GoGn (°)	33.27	1.84	38.03	1.65	<0.001*
ANS-Me (mm)	66.54	3.41	65.83	3.24	0.406
**Dental**					
SN^U1 (°)	105.72	6.25	102.93	5.53	0.071
SN^2M (°)	60.92	5.73	59.31	5.31	0.258
PTV-U1C (mm)	52.69	3.76	53.15	3.92	0.639
PTV-2MC (mm)	10.44	3.01	10.16	3.03	0.712
PP-U1T (mm)	28.04	1.74	27.27	2.67	0.166
PP-2MC (mm)	12.69	2.65	11.98	2.20	0.266
OVJ (mm)	7.75	1.63	7.88	1.41	0.745
OVB (mm)	1.97	2.61	0.68	2.41	0.049*
**Soft tissue**					
ULip-EPlane (mm)	−0.83	2.23	0.92	3.21	0.013*
LLip-EPlane (mm)	−0.11	2.33	1.57	2.86	0.012*

**Note:**

* *p* < 0.05.

### Intragroup changes of Group N between T0 and T1

At T1 (after treatment) significant changes were detected in most of the skeletal values as shown in Table 2. SNA, ANB, and ANS-Me changed significantly during treatment, −2.34 ± 1.95°, −1.95 ± 1–65, and 4.2 ± 2.3 mm, respectively (*p* < 0.01). SN^GoGn increased significantly during treatment by 0.76 ± 1.99° (*p* = 0.02). In accordance with the expected results, significant changes were detected for all dental and soft tissue measurements (*p* < 0.01). SN^U1 decreased 2.77 ± 6.36° while SN^2M increased 9.57 ± 6.95; PP-U1T and PP-2MC increased by 1.09 ± 1.52 mm and 3.2 ± 2.86 mm, respectively; OVJ and OVB decreased by 4.86 ± 1.62 mm and 1.55 ± 2.41 mm, respectively.

**Table 2:** Normodivergent intragroup comparison between T0 and T1.

	T0		T1		$\Delta$ T0–T1		95% CI	*p*
Measurement	Mean	SD	Mean	SD	Mean	SD		
**Skeletal**								
SNA (°)	83.04	3.16	80.70	3.50	−2.34	1.95	[−2.96 to −1.72]	<0.001*
SNB (°)	75.58	2.83	75.18	3.50	−0.39	1.34	[−0.82 to 0.04]	0.08
ANB (°)	7.47	1.84	5.52	2.01	−1.95	1.65	[−2.47 to −1.43]	<0.001*
SN^GoGn (°)	33.27	1.84	34.03	2.66	0.76	1.99	[0.13–1.39]	0.02*
ANS-Me (mm)	66.54	3.41	70.74	4.19	4.2	2.3	[3.47–4.93]	<0.001*
**Dental**								
SN^U1 (°)	105.72	6.25	102.95	4.50	−2.77	6.36	[−4.79 to −0.75]	0.01*
SN^2M (°)	60.92	5.73	70.49	5.51	9.57	6.95	[7.36–11.78]	<0.001*
PTV-U1C (mm)	52.69	3.76	50.00	3.24	−2.7	2.58	[−3.52 to −1.88]	<0.001*
PTV-2MC (mm)	10.44	3.01	17.10	5.74	6.66	5	[5.07–8.25]	<0.001*
PP-U1T (mm)	28.04	1.74	29.13	1.95	1.09	1.52	[0.61–1.57]	<0.001*
PP-2MC (mm)	12.69	2.65	15.89	2.37	3.2	2.86	[2.29–4.11]	<0.001*
OVJ (mm)	7.75	1.63	2.89	0.78	−4.86	1.62	[−5.38 to −4.34]	<0.001*
OVB (mm)	1.97	2.61	0.42	0.87	−1.55	2.41	[−2.32 to −0.78]	<0.001*
**Soft tissue**								
ULip-EPlane (mm)	−0.83	2.23	−3.81	2.21	−2.98	1.65	[−3.50 to −2.46]	<0.001*
LLip-EPlane (mm)	−0.11	2.33	−2.06	2.32	−1.95	1.91	[−2.56 to −1.34]	<0.001*

**Note:**

* *p* < 0.05.

### Intragroup changes of Group H between T0 and T1

Table 3 shows the changes during treatment for Group H. Significant changes were found for most variables. Among skeletal parameters, SNA and ANB decreased significantly by 2.29 ± 1.39° and 1.97 ± 1.21°, respectively meanwhile ANS-Me increased by 3.09 ± 2.37 mm (*p* < 0.01). Most dental and soft tissue measurements significantly changed between T0 and T1 (*p* < 0.01): SN^2M increased, indicating that the second maxillary molar tipped mesially by 7.74 ± 5.82°; PP-U1T and PP-2MC increased, indicating that the maxillary central incisor and second molar extruded by 0.95 ± 1.56 mm and 3.25 ± 1.5 mm, respectively; the OVJ was reduced by 5.13 ± 1.34 mm after treatment.

**Table 3:** Hyperdivergent intragroup comparison between T0 and T1.

	T0		T1		$\Delta$ T0–T1		95% CI	*p*
Measurement	Mean	SD	Mean	SD	Mean	SD		
**Skeletal**								
SNA (°)	81.62	2.53	79.33	2.68	−2.29	1.39	[−2.85 to −1.73]	<0.001*
SNB (°)	73.46	2.15	73.14	2.21	−0.32	0.91	[−0.69 to 0.05]	0.08
ANB (°)	8.17	2.04	6.2	2.42	−1.97	1.21	[−2.46 to −1.48]	<0.001*
SN^GoGn (°)	38.03	1.65	37.8	2.92	−0.23	2.25	[−1.14 to 0.68]	0.60
ANS-Me (mm)	65.83	3.24	68.92	4.2	3.09	2.37	[2.13–4.05]	<0.001*
**Dental**								
SN^U1 (°)	102.93	5.53	101.33	4.07	−1.59	6.94	[−4.39 to 1.21]	0.250
SN^2M (°)	59.31	5.31	67.05	6.11	7.74	5.82	[5.39–10.09]	<0.001*
PTV-U1C (mm)	53.15	3.92	49.86	3.26	−3.3	3.08	[−4.54 to −2.06]	<0.001*
PTV-2MC (mm)	10.16	3.03	17.34	4.57	7.18	3.61	[5.72–8.64]	<0.001*
PP-U1T (mm)	27.27	2.67	28.21	2.26	0.95	1.56	[0.32–1.58]	<0.001*
PP-2MC (mm)	11.98	2.2	15.23	2.22	3.25	1.5	[0.91–3.53]	<0.001*
OVJ (mm)	7.88	1.41	2.75	0.87	−5.13	1.34	[−5.67 to −4.59]	<0.001*
OVB (mm)	0.68	2.41	0.27	0.76	−0.4	2.29	[−1.33 to 0.53]	0.38
**Soft tissue**								
ULip-EPlane (mm)	0.92	3.21	−2.25	2.9	−3.17	1.57	[−3.80 to −2.54]	<0.001*
LLip-EPlane (mm)	1.57	2.86	−0.6	2.84	−2.17	1.78	[−2.89 to −1.45]	<0.001*

**Note:**

* *p* < 0.05.

### Intergroup changes between Group N and Group H at T1

Even though some differences were detected for skeletal, dental, and soft tissue variables, none of them reached the level of significance as reported in Table 4 (*p* > 0.05). All measurements changed into the same direction between groups except for SN^GoGn; in Group N this angle increased by 0.76 ± 1.99 mm while in Group H it decreased by 0.23 ± 2.25 mm, but these values were not statistically significant nor clinically relevant.

**Table 4:** Intergroup comparison between normodivergent and hyperdivergent group at $\Delta$ T0–T1.

	Normodivergent $\Delta$ T0–T1		Hyperdivergent $\Delta$ T0–T1		*p*
Measurement	Mean	SD	Mean	SD	
**Skeletal**					
SNA (°)	−2.34	1.95	−2.29	1.39	0.900
SNB (°)	−0.39	1.34	−0.32	0.91	0.813
ANB (°)	−1.95	1.65	−1.97	1.21	0.973
SN^GoGn (°)	0.76	1.99	−0.23	2.25	0.680
ANS-Me (mm)	4.2	2.3	3.09	2.37	0.066
**Dental**					
SN^U1 (°)	−2.77	6.36	−1.59	6.94	0.485
SN^2M (°)	9.57	6.95	7.74	5.82	0.276
PTV-U1C (mm)	−2.7	2.58	−3.3	3.08	0.403
PTV-2MC (mm)	6.66	5.00	7.18	3.61	0.645
PP-U1T (mm)	1.09	1.52	0.95	1.56	0.268
PP-2MC (mm)	3.2	2.86	3.25	1.5	0.303
OVJ (mm)	−4.86	1.62	−5.13	1.34	0.479
OVB (mm)	−1.55	2.41	−0.4	2.29	0.061
**Soft tissue**					
ULip-EPlane (mm)	−2.98	1.65	−3.17	1.57	0.655
LLip-EPlane (mm)	−1.95	1.91	−2.17	1.78	0.639

## Discussion

This cephalometric outcome study revealed that extraction of maxillary first molars followed by fixed appliance therapy in normo- and hyperdivergent patients with a Class II division 1 malocclusion leads to comparable changes for skeletal, dento-alveolar and soft tissue profile variables in both facial types. All patients ended with a Class I molar relationship (upper 7th to lower 6th) at the end of treatment and a large improvement of the occlusion. Since the treatment was carried out in growing individuals and an untreated control group was not available, it was not possible to discriminate between potential growth-related and therapeutic effects.

The skeletal changes during treatment were not significantly different between both facial patterns. ANB values decreased similarly in both groups (Group N: −1.95 ± 1.65°; Group H: −1.97 ± 1.21°) but it was observed that this change was mainly caused by a concordant reduction of the SNA angle (Group N: −2.34 ± 1.95°; Group H: −2.29 ± 1.39°). Mandibular growth was clinically relevant as demonstrated by the SNB angle that remained unchanged despite the increase of the anterior facial dimension; The post-treatment unchanged ANB angle was probably due to a combination of mandibular clockwise rotation suggested by the increase of ANS-Me (Group N: 4.2 ± 2.3 mm; Group H: 3.09 ± 2.37 mm) (Booij et al., 2022) and a compensation produced by the increase in mandibular length due to growth. Finally, changes in divergency at T1 were statistically relevant only for Group N but not between groups; this finding suggests that anterior facial height was also balanced in the posterior one through increase in the dimension of mandibular ramus.

After treatment the cephalometric position of the maxillary second molar showed that a mesial bodily movement (Group N: 6.66 ± 5 mm; Group H: 7.18 ± 3.61 mm) and a mesial tipping (Group N: 9.57 ± 6.95°; Group H: 7.74 ± 5.82°) had occurred whereas the upper incisors showed a distal bodily movement (Group N: −2.7 ± 2.58 mm; Group H: −3.3 ± 3.08 mm) and a crown distal tipping (Group N: −2.77 ± 6.36°; Group H: −1.59 ± 6.94°).Small differences between groups may be explained by the fact that a significant relationship exists between facial muscle activity and facial growth pattern. The masticatory muscle activity has been proven being significantly lower in patients with a vertical facial pattern compared with other growth patterns (Alabdullah et al., 2015); therefore, in the hyperdivergent patients muscle forces may be lower and this could be the reason of differences between bodily movement and crown tipping of this facial type compared to normodivergent ones. Vertically, maxillary second molars and incisors showed a significant extrusion in both groups after treatment; this fact may explain the increase of anterior facial height even all vertical changes between groups were not statistically significant.

In both groups the OVJ was reduced significantly during treatment (Group N: −4.86 ± 1.62 mm; Group H: −5.13 ± 1.34 mm); In both facial types this reduction is primarily achieved by an upper incisor retrusion and in accordance with the biomechanics used for Class II correction. The OVB was slightly larger in the normodivergent group at T0 which was to be expected as overbite was correlated to the selection parameters used for the division of our sample into normo- and hyperdivergent facial type; despite that, changes in OVB during treatment were clinically irrelevant in both groups.

It is known that orthodontic treatment also affects the soft tissue and anatomical structures such as the nasal region (Janson et al., 2016; Konstantonis et al., 2018; Moser et al., 2020). In both facial types the upper and lower lips were in a more retruded position at the end of treatment. These changes should be understood in relation to normal growth changes during this period. It has been shown that lips become significantly more retruded with respect to the E-line over time (Bishara et al., 1998; Nanda et al., 1990). Ricketts, 1968 suggested that ideally the upper lip should be 4 mm posterior to E-line in women and slightly more retruded in men, and the lower lip should ideally be 2 mm posterior to the E-line in women and slightly more behind in man. However, in another study from our research group no sex differences were found for sagittal and vertical soft tissue measurements (Zecca et al., 2016). At the start of treatment, the upper lip (Group N: −0.83 ± 2.23 mm; Group H: 0.92 ± 3.21 mm) and lower lip (Group N: −0.11 ± 2.33 mm; Group H: 1.57 ± 2.86 mm) were protruded in both groups, whereas after treatment the values for the position of the upper and lower lip in relation to E-line were comparable to the values reported by Ricketts (1968). Even while the mean values at T0 of ULip to EPlane and LLip to EPlane between Group N and Group H showed statistically significant differences, the mean amount of retrusion of the upper and lower lips in relation to E-Line was similar in both groups and resulted in an improvement of facial profile. Indeed, after treatment, lip position values were close to the ideal values suggested by Ricketts for normal and untreated adults of similar age (Denize et al., 2014).

The control of the vertical dimension has always been a challenge for orthodontists because in hyperdivergent individuals there is a risk that the divergency increases due to the orthodontic treatment and the unfavorable facial growth pattern. A recent systematic review suggested, however, that orthodontic treatment including premolar extractions has no clear effect on the skeletal vertical dimension (Kouvelis et al., 2018). In fact, posterior tooth extractions, *i.e*., second premolars or first molars, have been suggested to better control the vertical dimension, whereas additionally, forward rotation of the mandible may improve the Class II (Garlington & Logan, 1990). On the other hand, many studies reported no distinct effects of extractive therapy on the facial vertical dimension as was shown in the present one.

The previously mentioned “wedge-type effect” is responsible for overclosure of the mandible resulting in a decrease in vertical dimension or control of it (Kim et al., 2005). For the same mechanical reason, maxillary first molar extraction should guarantee a larger mesial movement of posterior teeth compared to premolar extraction and the vertical dimension must be theoretically lowered at the end of treatment. This hypothesis was rejected by the present results, also when premolar and molar extractions for Class II malocclusion treatment are compared (Booij et al., 2022). Probably, the main bias to exclude when attempting to better understand any potential correlation, is the residual mandibular vertical growth that can undo any treatment improvement; also, the use of fixed orthodontic appliances is related to an increase in vertical dimension by teeth extrusion during interarch mechanics and clockwise rotation of the mandible (Chhibber et al., 2011), and it may explain the fact that in both normo- and hyperdivergent patients the vertical dimension slightly changed at the end of the treatment. The extrusion of posterior teeth tends to keep step with the vertical increase in facial height, maintaining the occlusal plane tilting and nullifying any closing effect (Upadhyay et al., 2008). Furthermore, neither the extraction of four first molars is effective in reducing the vertical dimension (Hans et al., 2006); for these reasons, vertical control is a complex issue to perform, especially in growing patients.

Finally, the current study shows that extraction of maxillary first molars could be a viable treatment option in patients with a Class II division 1 malocclusion both in normo- and hyperdivergent facial types regarding the vertical dimensions and the Class II correction; however, a treatment protocol for maxillary molar extractions specifically aiming at reduction or control of the vertical dimension does not seem to be an evidence-based clinical approach.

The main limitations of this study are related to the study design; this is a single center, single operator, and retrospective study that limits the generalizability of the results and selection bias cannot be ruled out. This was a longitudinal study without a control group. Therefore, we cannot differentiate between growth changes and treatment changes. There are hardly any studies in the literature that consider maxillary first molar extraction. For that reason, it was difficult to calculate a proper *a priori* sample size. Finally, the use of 2D cephalograms can be a limitation. 3D analysis especially of the soft tissues should be promoted in further studies.

## Conclusions

The results of the present study indicated that orthodontic treatment with fixed appliances including extraction of maxillary first molars in growing individuals is successful in correcting Class II division I malocclusion and improving the profile in patients with a hyperdivergent and normodivergent facial type. The extraction of the maxillary first molars does not reduce the skeletal divergency of growing patients even though the posterior dental fulcrum might be affected, especially in hyperdivergent facial type. Therefore, this orthodontic approach can be considered a viable option in the armamentarium of the Class II Division I therapy for both facial types.

## Supplemental Information

10.7717/peerj.14537/supp-1 Supplemental Information 1 Raw data.

## Additional Information and Declarations

### Data Availability

The following information was supplied regarding data availability: The deidentified raw data are available in the Supplemental File.
